# Influences of Yogurt with Functional Ingredients from Various Sources That Help Treat Leaky Gut on Intestinal Barrier Dysfunction in Caco-2 Cells

**DOI:** 10.3390/ph16111511

**Published:** 2023-10-24

**Authors:** Ricardo S. Aleman, Ryan Page, Roberto Cedillos, Ismael Montero-Fernández, Jhunior Abraham Marcia Fuentes, Douglas W. Olson, Kayanush Aryana

**Affiliations:** 1 School of Nutrition and Food Sciences, Louisiana State University Agricultural Center, Baton Rouge, LA 70802, USA; rsantosaleman@lsu.edu (R.S.A.); rpage1@lsu.edu (R.P.); rcedil1@lsu.edu (R.C.); dolson@agcenter.lsu.edu (D.W.O.); 2 Department of Plant Biology, Ecology and Earth Sciencies, Faculty of Science, Universidad de Extremadura, Avda. de Elvas s/n, 06071 Badajoz, Spain; ismonterof@unex.es; 3 Faculty of Technological Sciences, Universidad Nacional de Agricultura, Road to Dulce Nombre de Culmí, Km 215, Barrio El Espino, Catacamas 16201, Honduras; jmarcia@unag.edu.hn

**Keywords:** Caco-2 cells, tight junctions, leaky gut ingredients, yogurt, intestinal barrier dysfunction

## Abstract

The impact of yogurts made with starter culture bacteria (*L. bulgaricus* and *S. thermophilus*) and supplemented with ingredients (maitake mushrooms, quercetin, L-glutamine, slippery elm bark, licorice root, N-acetyl-D-glucosamine, zinc orotate, and marshmallow root) that can help treat leaky gut were investigated using the Caco-2 cell monolayer as a measure of intestinal barrier dysfunction. Milk from the same source was equally dispersed into nine pails, and the eight ingredients were randomly allocated to the eight pails. The control had no ingredients. The Caco-2 cells were treated with isoflavone genistein (negative control) and growth media (positive control). Inflammation was stimulated using an inflammatory cocktail of cytokines (interferon-γ, tumor necrosis factor-α, and interleukin-1β) and lipopolysaccharide. The yogurt without ingredients (control yogurt) was compared to the yogurt treatments (yogurts with ingredients) that help treat leaky gut. Transepithelial electrical resistance (TEER) and paracellular permeability were measured to evaluate the integrity of the Caco-2 monolayer. Transmission electron microscopy (TEM), immunofluorescence microscopy (IM), and real-time quantitative polymerase chain reaction (RTQPCR) were applied to measure the integrity of tight junction proteins. The yogurts were subjected to gastric and intestinal digestion, and TEER was recorded. Ferrous ion chelating activity, ferric reducing potential, and DPPH radical scavenging were also examined to determine the yogurts’ antioxidant capacity. Yogurt with quercetin and marshmallow root improved the antioxidant activity and TEER and had the lowest permeability in fluorescein isothiocyanate (FITC)–dextran and Lucifer yellow flux among the yogurt samples. TEM, IM, and RTQPCR revealed that yogurt enhanced tight junction proteins’ localization and gene expression. Intestinal digestion of the yogurt negatively impacted inflammation-induced Caco-2 barrier dysfunction, while yogurt with quercetin, marshmallow root, maitake mushroom, and licorice root had the highest TEER values compared to the control yogurt. Yogurt fortification with quercetin, marshmallow root, maitake mushroom, and licorice root may improve functionality when dealing with intestinal barrier dysfunction.

## 1. Introduction

The gastrointestinal (GI) tract has a complex tissue structure that allows it to develop a great diversity of physiological processes, such as digestion, the absorption of nutrients, and the secretion of water and electrolytes, as well as motor functions for the regulating gastrointestinal contents and broad immunological activity. Histologically, the entire GI tract has a similar common structure, consisting of four concentric layers from the outside to the intestinal lumen. All the functions carried out by the GI tract require fine regulation, and specific nervous and endocrine mechanisms exist. For these reasons, it is important to develop strategic approaches that could assist the gastrointestinal (GI) tract in improving its functions and reducing its dysfunction. Gastrointestinal disorders are among the health problems that affect the population with the greatest frequency regardless of age and socioeconomic level. These disorders are related to other combinations of motility disorders, visceral hypersensitivity, intestinal microbiota alteration, and nervous system disorders [1,2,3]. Furthermore, these disorders are related to allergies and affect the gastrointestinal tract (GIT), with abundant inflammation in eosinophils. The incidence of gastrointestinal diseases is increasing in Western countries [3]. Leaky gut syndrome is a poorly understood disorder characterized by patients presenting with headaches, fatigue, diarrhea, abdominal swelling, food intolerance, difficulty losing weight, and joint pain without an apparent cause. Leaky gut is related to pathogenesis such as obesity, celiac disease, diabetes, asthma, inflammatory bowel disease, and multiple sclerosis [4]. At least 100 million people have leaky guts [5]. As a result, yogurt fortified with functional ingredients has been carefully investigated to help treat gastrointestinal disorders such as diarrhea, irritable bowel syndrome, nausea, and bloating. Likewise, probiotics can also reduce dysfunction in the intestinal barrier caused by cytokines. Pretreatment of the intestinal epithelium with *S. thermophilus* and *L. acidophilus* or with the commensal bacteria *Bacteroides thetaiotaomicron* is shown to prevent resistance decreased due to transepithelial electrical activity caused by TNF-α and IFN-γ [2]. On the other hand, recent studies showed that the administration of the probiotic VSL#3 prevents the reduction in and redistribution of ZO-1, occludin, and claudin 1, 3, 4, and 5 [2].

Yogurt is considered a functional food since it is a good source of vitamins B12, folic acid, potassium, magnesium, zinc, calcium, and phosphorus; it contains elements that contribute to maintaining or improving the gut and metabolism. The benefits of yogurt are related to the matrix’s effect rather than the impact of nutrients on their own [6]. Some whey proteins, β-lactoglobulin and α-lactalbumin, interact with minerals such as calcium, favoring its absorption [7]. Traditionally, yogurt consumption’s best-known and evidenced effects have been related to GIT health benefits. Yogurt can improve symptoms of acute diarrhea in children [8], as well as certain immuno-inflammatory diseases, such as allergies [9] and inflammatory bowel disease (IBD) [10]. Epidemiological studies have revealed an association between the consumption of yogurt and benefits at the metabolic level, such as the control of type 2 diabetes [11] and the improvement of body weight and adiposity [12]. At the same time, its effects on cardiovascular disease risk markers and the lipid profile appear neutral [13,14].

Yogurt has been related to anti-inflammatory activity in metabolic conditions, including overweight disorders [15]. Meng et al. (2017) [16] demonstrated that yogurt enhances TLR-2 in monocytic cells but does not change the IL-6 or TNF-α levels in peripheral blood mononuclear cells. On the other hand, Meyer et al. (2007) [17] presented that yogurt increases the levels of IL-1β and TNF-α in PBMC induced by phytohemagglutinin or LPS. Pei et al. (2017) [18] demonstrate a decrease in TNF-α plasma levels and the enhancement of LPS-binding protein (LBP)/sCD14 markers when compared to the control group. N-acetyl-D-glucosamine (NAG), zinc orotate (ZN), quercetin (Q), L-glutamine (LG), slippery elm bark (SEB), marshmallow root (MR), licorice root (LR), and maitake mushrooms (MM) have shown potential benefits through improving the intestinal barrier.

Licorice root and NAG have been demonstrated to reduce pro-inflammatory cytokine levels [19,20], whereas slippery elm bark, quercetin, and L-glutamine can help strengthen tight junctions [21,22,23]. Zinc orotate and marshmallow root presented anti-inflammatory and antioxidant characteristics [24,25] and decreased intestinal permeability. Furthermore, maitake mushrooms contain significant amounts of beta-1,6-glucans, an immune stimulant [26].

As far as yogurt is concerned, its production derives from the symbiosis between two bacteria, *Streptococcus thermophilus* and *Lactobacillus bulgaricus*, which are characterized by the fact that each one stimulates the development of the other. This interaction considerably reduces fermentation time, and the resulting product has peculiarities that distinguish it from those fermented by a single or different strain of bacteria, which could be called fermented milk [27].

Consuming yogurt can help the intestine develop a balanced microbiota favorable to intestinal health. This food product helps to maintain the proper functioning of the intestine. Low-fat yogurt can modulate cytokine IL-1β in Caco-2 cells [28]. Consequently, it is important to fortify yogurt with functional ingredients that could help improve intestinal barrier functions.

In other words, yogurt fortified with these ingredients can help improve intestinal barrier integrity. The hypothesis is that if these ingredients have nutrients that can help treat leaky gut, they may have the potential to differentially influence intestinal barrier dysfunction, as can be determined using Caco-2 cells. Our objective was to study the effects of yogurt fortified with these ingredients on intestinal permeability using the Caco-2 cell model.

## 2. Results and Discussion

### 2.1. Antioxidant Capacity of Yogurt with Functional Ingredients

The variation in antioxidant capacity in yogurt can be influenced by various factors, such as the source or type of fruit or plant material from which it is extracted. Furthermore, the daily intake of polyphenols differed significantly between individuals (183–4854 mg/day); therefore, fortifying yogurt with phenol-rich ingredients is crucial [29]. The DPPH radical scavenging activity, ferric reducing antioxidant potential (FRAP) activity, and ferrous ion chelating (FIC) activity of fortified yogurts with functional ingredients are shown in Table 1. For the DPPH radical scavenging and ferrous ion chelating, the antioxidant activity of yogurt supplemented with quercetin and marshmallow root was significantly (*p* < 0.05) higher than that of the control yogurt and the remaining treatments. Marshmallow root polysaccharides have shown high antioxidant properties [29].

Quercetin is a distinctive flavonoid that is commonly found in vegetables and fruits, and its application has demonstrated remarkable antioxidant capacity in vivo [30]. For FRAP, only the antioxidant activity of yogurt with quercetin was significantly (*p* < 0.05) higher than that of the control yogurt. The DPPH radical scavenging activity and FRAP values for quercetin and marshmallow root samples had a similar tend, having the highest values for both assays (Table 1).

The observation that FRAP had more significant differences among treatments compared to significant the differences among treatments for DPPH and FIC may be due to the diverse phenolic profile in Marshmallow root, affecting their hydrogen or electron transfer capacity to reduce ferric tripiridyltriazine to the ferrous form (Fe^++^) at low pH [31]. Ferreira and Santos (2023) [32] studied the effect of incorporating agro-industrial by-products of fruits with a high concentration of phenolic compounds, such as chestnuts, grape seeds, or pomegranate, in fortified yogurts, and all demonstrated antioxidant and antimicrobial properties, finding antioxidant capacity values of 1128 and 972 mg Trolox g^−1^ fraction extracts for the DPPH and ABTS assays, respectively. Plants produce large amounts of secondary metabolites to better adapt to environmental conditions, protect themselves from microbial attacks, and resist both biotic and abiotic stress. These phenolic compounds have received significant attention recently due to their antioxidant, anti-inflammatory, and anticoagulant properties, correlated with a decreased risk of cardiovascular diseases and cancer development.

### 2.2. Caco-2 Cell Viability

**Figure 1:**
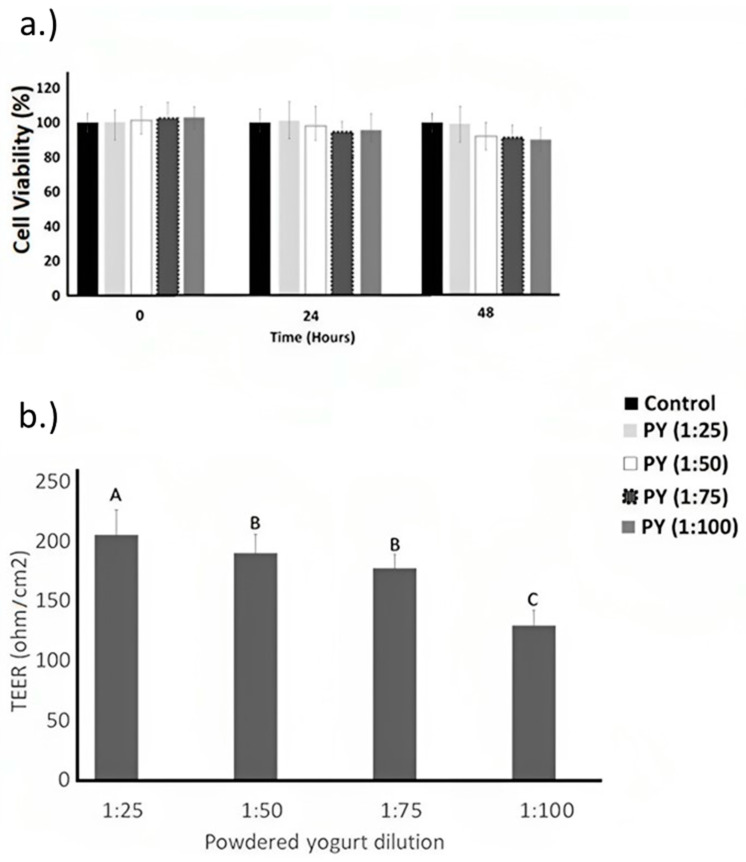
(**a**) Caco-2 cell proliferation during incubation with different ratios of yogurt powder diluted with water. There were no significant (*p* < 0.05) differences among treatments or over time in one-way ANOVA. Control yogurt = PY. Caco-2 cells treated with phosphate-buffered saline (PBS) were used as negative control (black bars). (**b**) Effect of powdered yogurt dilutions on barrier function for differentiated Caco-2. Powdered yogurt was resuspended in growth media at 1:25, 1:50, 1:75, and 1:100 dilutions before application to cell monolayers. TEER was measured after 48 h. ^ABC^ Different letters indicate significant (*p* < 0.05) differences among treatments for TEER values in one-way ANOVA followed by Tukey test.

The evaluation of Caco-2 cells using in vitro models of small intestinal integrity has been carried out by Putt et al. (2017). This type of cell can mimic the absorption of nutrients and form a tight barrier against potentially harmful components such as pro-inflammatory cytokines. The cell viability and transepithelial electrical resistance (TEER) of Caco-2 cells, as influenced by the yogurt dilutions (1:25, 1:50, 1:75, and 1:100) over 48 h of incubation, are shown in Figure 1A,B, respectively. For cell viability, the dilution concentration, time effect, and dilution concentration × time interaction effect were insignificant (*p* > 0.05). Compared to the control samples (Caco-2 cells treated with phosphate-buffered saline), no significant difference was observed in cell viability after 48 h of incubation for all yogurt powders (1:25, 1:50, 1:75, and 1:100) (Figure 1A). Yogurt did not affect Caco-2 cells in the apoptotic pathway. On the other hand, yogurt powder dilution at 1:25 had the highest transepithelial electrical resistance compared to the other yogurt dilutions (1:50, 1:75, and 1:100) (Figure 1B).

Putt et al. (2017) [33] reported higher transepithelial electrical resistance in Caco-2 cells using a 1:30 low-fat yogurt dilution compared to powdered yogurt resuspended in growth media at 1:25, 1:50, and 1:100 dilutions. TEER examines the permeability and integrity of Caco-2 cells [34]. TEER indicates the rate of ionic conductance of the paracellular route in the epithelial barrier, and with higher TEER, the integrity of Caco-2 cells is more preserved [35]. As a result, a 1:25 yogurt dilution was used for subsequent tests for Caco-2 cells. The Caco-2 cell model is used to evaluate gastrointestinal permeability in vitro. The results obtained with this cell line show both successes and failures in its predictive capacity and indicate that factors such as the source or origin of the cells and the passage number, among others, cause variability in the permeability results for the same compound. It is important to test the viability of the caco2 cells when adding yogurt.

### 2.3. TEER and Paracellular Permeability Observations

**Figure 2:**
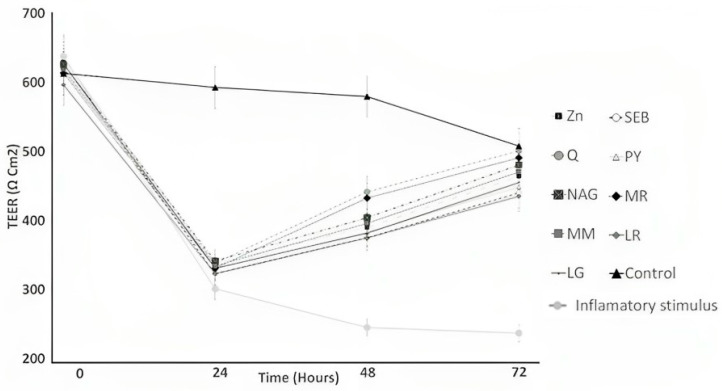
Yogurt samples (Ys) raise the TEER of Caco-2 cell monolayers exposed to an inflammatory stimulus. Caco-2 cells were treated with a control (growth media), and an inflammatory stimulus (I) consisting of interleukin-1β (IL-1β, 25 ng mL^−1^), tumor necrosis factor-α (TNF-α, 50 ng mL^−1^), and interferon-gamma (IFN-γ, 50 ng mL^−1^) or I and Ys from 0 to 72 h. Zn = zinc orotate, Q = quercetin, NAG = N-acetyl-D-glucosamine, MM = maitake mushrooms, LG = L-glutamine, SEB = slippery elm bark, PY = control Yogurt, MR = marshmallow root, and LR = licorice root.

The TEER of yogurt samples, as influenced by the incorporation of the ingredients over 72 h of incubation, is illustrated in Figure 2. The ingredient effect, time effect, and ingredient × time interaction effect were significant (*p* < 0.05). With cytokine exposure to cells, the TEER significantly increased from 48 to 72 h for yogurt samples incorporating quercetin and marshmallow root compared to the control yogurt samples (Figure 2). The TEER of the control yogurt samples did not show a decline like the cells treated only with the mixture at induction from 0 to 24 h, and the significant increased between 24 and 48 h (Figure 2). TEER allows us to evaluate the level of expression of intercellular junctions indirectly, and the most decisive structural factors related to changes in the values of this parameter are the profile and levels of the different tight junctions expressed by the cell line.

Quercetin and marshmallow root phenolic content could help to improve induced barrier functions [36,37]. Polyphenols’ mechanisms of action in improving intestinal barrier dysfunction are not fully comprehended. However, polyphenols are implicated in NF-κB inactivation, which is related to upregulating tight junctions. In addition, the NF-κB inactivation pathway is recognized as one of the most influential in regulating pro-inflammatory and anti-inflammatory markers [2].

**Figure 3:**
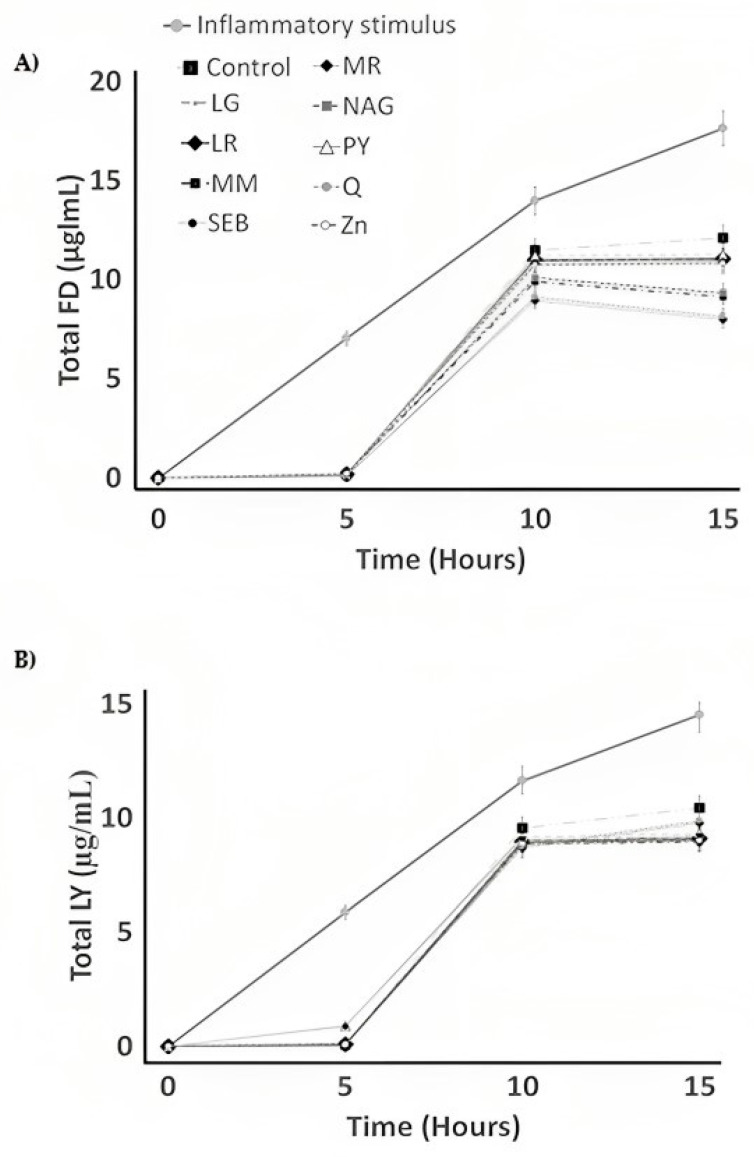
The flux of (**A**) fluorescein isothiocyanate–dextran (FD) and (**B**) Lucifer yellow (LY) in differentiated Caco-2 cells exposed to vehicle control (C), inflammatory stimulus (I), or inflammatory stimulus and CYs for 15 h. LG = L-glutamine, LR = licorice root, MM = maitake mushrooms, SEB = slippery elm bark, MR = marshmallow root, NAG = N-acetyl-D-glucosamine, PY = control yogurt, Q = quercetin, and Zn = zinc orotate.

TEER is commonly measured to verify the Caco-2 barrier function in a paracellular permeability assay. Compared to the control yogurt samples, a significant (*p* < 0.05) decrease in fluorescein isothiocyanate (FITC)–dextran (FD) flux was observed for yogurt samples made with quercetin and marshmallow root (Table 2; Figure 3A). On the other hand, no significant difference (*p* > 0.05) in Lucifer yellow (LY) flux was observed for yogurt samples made with different ingredients when compared to the control yogurt (Table 2; Figure 3B).

All yogurt samples (Q, MR, NAG, LG, ZN, MM, LR, SEB, and CY) significantly (*p* < 0.05) decreased the permeability of LY and FD when compared to cells treated with just inflammatory stimulus. Not surprisingly, the inflammatory stimulus is expected to harm the Caco-2 cell monolayer’s integrity [38]. To date, yogurt, quercetin, and marshmallow root have been shown to improve probiotic characteristics, which could impact intestinal barrier dysfunction [39]. Nevertheless, the mechanisms of action of yogurt, quercetin, and marshmallow root still need to be better understood, and more research on this topic is encouraged.

In vivo and in vitro studies have shown that flavonoids can modulate the activity of carcinogenic metabolites that are formed in the carcinogenic process. Quercetin exerts inhibitory effects against cancer cells in the colon, mammary gland, ovary, gastrointestinal regions, and leukemia. This could be due to the increase in intracellular glutathione concentrations. However, these compounds may present pro-oxidant effects. The molecular mechanisms that determine this activity are based on the formation of a labile aroxyl radical or a labile redox iron–flavonoid complex. In the first case, the autoxidation of the aroxyl radical generates a superoxide anion, which produces a harmful hydroxyl radical following the known sequence. These mechanisms may constitute the basis of the mutagenic and cytotoxic actions described for some flavonoids. These actions only seem to occur when the doses of flavonoids are very high [2].

### 2.4. Transmission Electron Microscopy (TEM)

**Figure 4:**
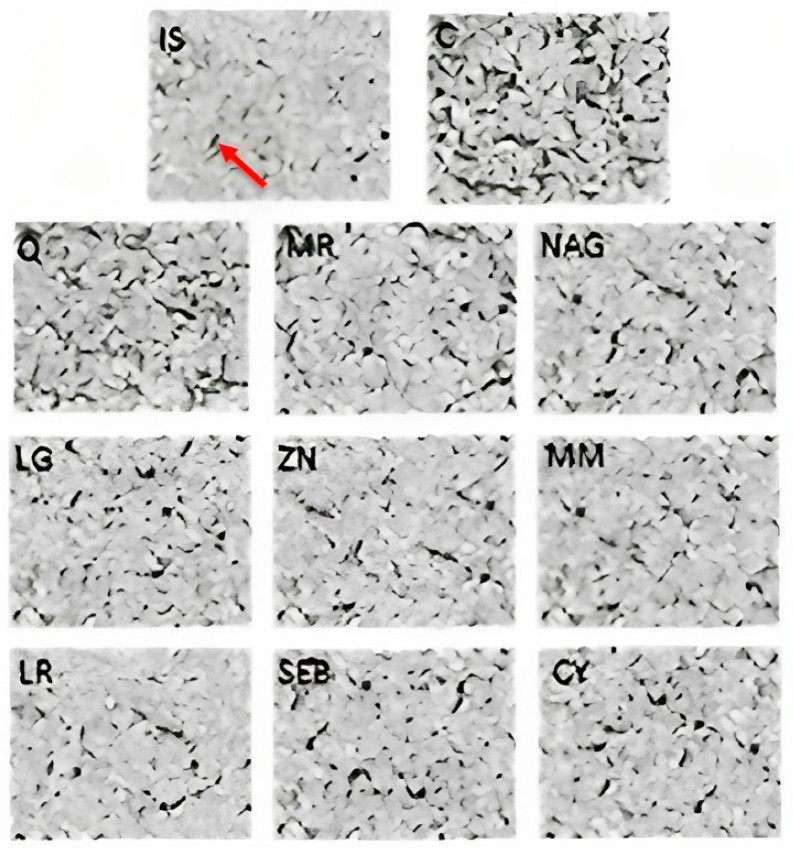
TEM micrographs of yogurt samples, inflammatory stimulus (IS), and growth media (C) in differentiated Caco-2 cells after 48 h. Pictures were taken under approximately 90 nm^2^. Yogurt samples: Q = quercetin, MR = marshmallow root, NAG = N-acetyl-D-glucosamine, LG = L-glutamine, ZN = zinc orotate, MM = maitake mushrooms, LR = licorice root, SEB = slippery elm bark, and CY = control yogurt. Red arrow indicates the black streaks (tight junctions).

Tight junctions constitute microdomains of the plasma membrane. These structures separate the apical from the basolateral domain of epithelial cells, generating extracellular compartments with different compositions. Consequently, epithelia and tight junctions act as a barrier to fluid diffusion between two compartments, with selective permeability to ions, growth factors, pathogens, and other solutes [2]. Transmission electron microscopy images of Caco-2 cells are shown in Figure 4. As shown by the red arrow (C), the electron-dense spots and black streak areas are the desmosomes and the tight junctions, as indicated in previous studies [40]. For the cells treated only with the mixture for induction, the desmosome and the tight junctions seem to be retained (Figure 4 (IS)), meaning a smaller area of black streaks was observed when compared to the cells treated with yogurt samples (Q, MR, NAG, LG, ZN, MM, LR, SEB, and CY). When the cells were treated with yogurt fortified with quercetin (Q), marshmallow root (MR), and N-acetyl-D-glucosamine (NAG), black streaks appeared to be more preserved when compared to the other yogurt samples (LG, ZN, MM, LR, SEB, and CY). TEM images showed similar results for the paracellular permeability and TEER measurements. Yogurt enhances gut barrier functions in Caco-2 cells by improving tight junctions [33].

### 2.5. Immunofluorescence Microscopy (IM)

**Figure 5:**
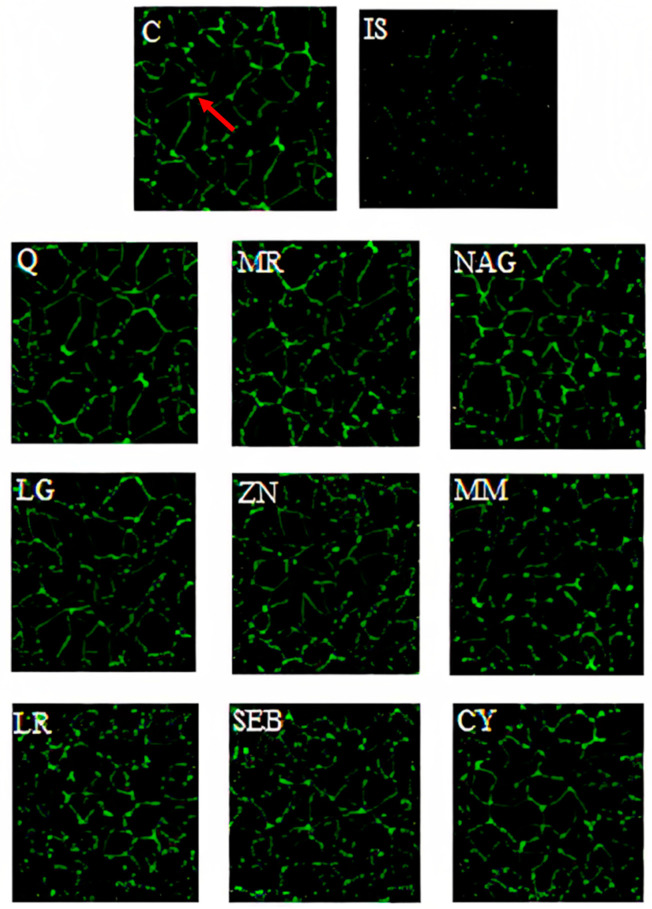
ZO-1 immunofluorescence microscopy pictures of yogurt samples, inflammatory stimulus (IS), and growth media (C) in differentiated Caco-2 cells after 48 h. Pictures are taken under approximately 70 nm^2^. Yogurt samples: Q = quercetin, MR = marshmallow root, NAG = N-acetyl-D-glucosamine, LG = L-glutamine, ZN = zinc orotate, MM = maitake mushrooms, LR = licorice root, SEB = slippery elm bark, and CY = control yogurt. Red arrow indicates the green pattern (ZO-1 tight junction).

**Figure 6:**
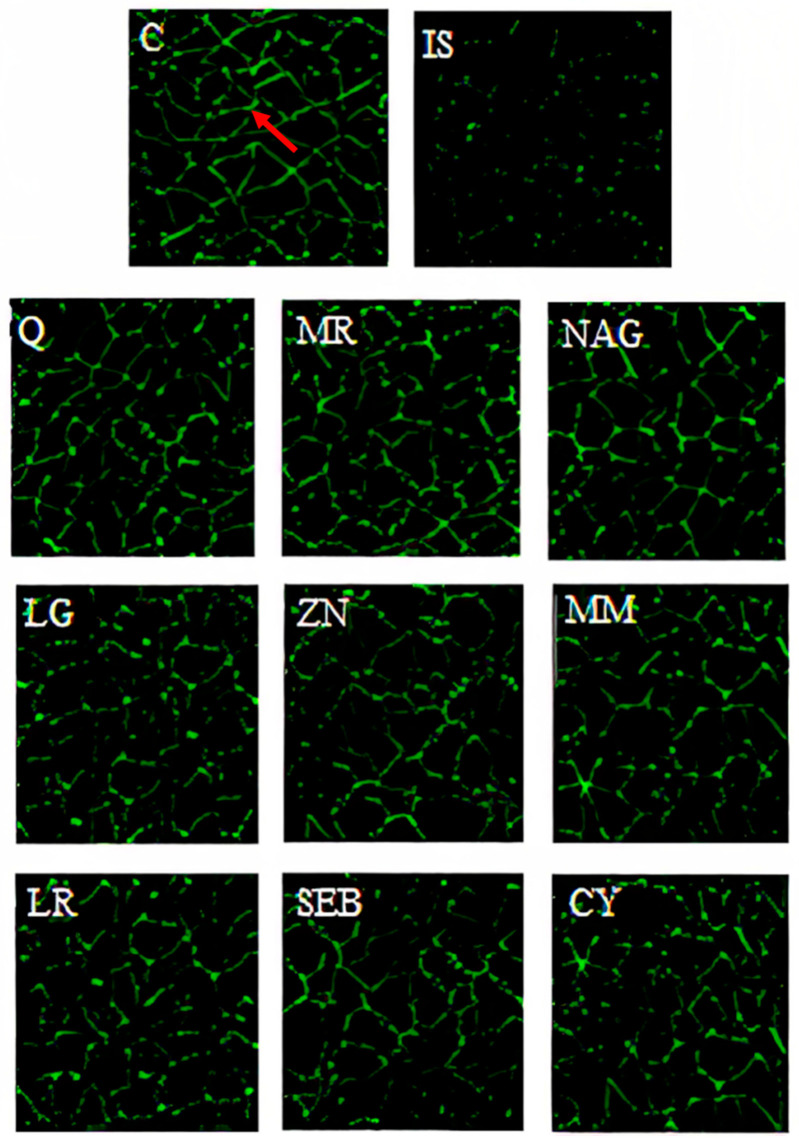
Occludin-1 immunofluorescence microscopy pictures of yogurt samples, inflammatory stimulus (IS), and growth media (C) in differentiated Caco-2 cells after 48 h. Pictures are taken under approximately 70 nm^2^. Yogurt samples: Q = quercetin, MR = marshmallow root, NAG = N-acetyl-D-glucosamine, LG = L-glutamine, ZN = zinc orotate, MM = maitake mushrooms, LR = licorice root, SEB = slippery elm bark, and CY = control yogurt. Red arrow indicates the green pattern (occludin-1 tight junction).

**Figure 7:**
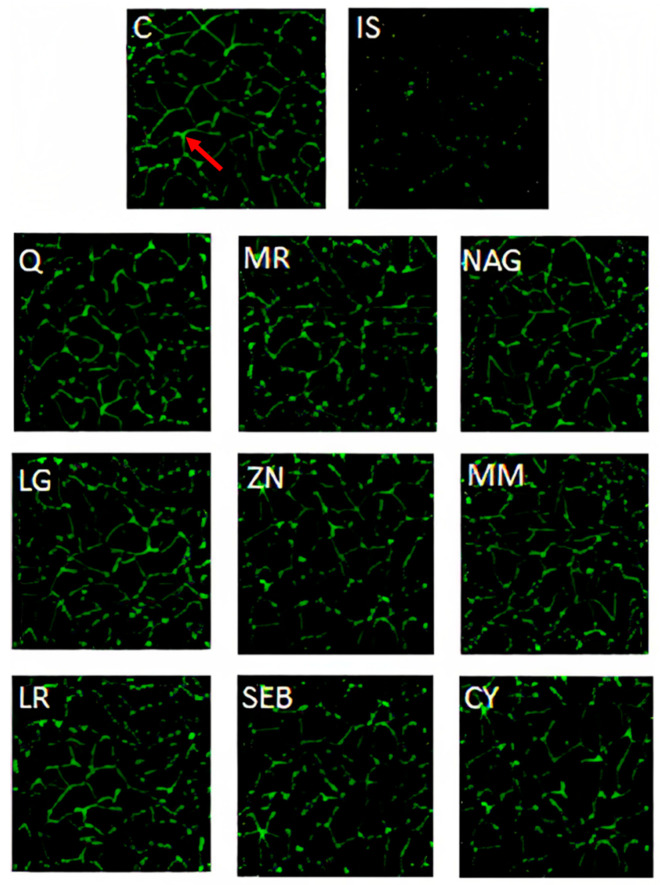
Immunofluorescence pictures of claudin-1 yogurt samples, inflammatory stimulus (IS), and growth media (C) in differentiated Caco-2 cells after 48 h. Pictures are taken under approximately 70 nm^2^. Yogurt samples: Q = quercetin, MR = marshmallow root, NAG = N-acetyl-D-glucosamine, LG = L-glutamine, ZN = zinc orotate, MM = maitake mushrooms, LR = licorice root, SEB = slippery elm bark, and CY = control yogurt. Red arrow indicates the green pattern (claudin-1 tight junction).

**Figure 8:**
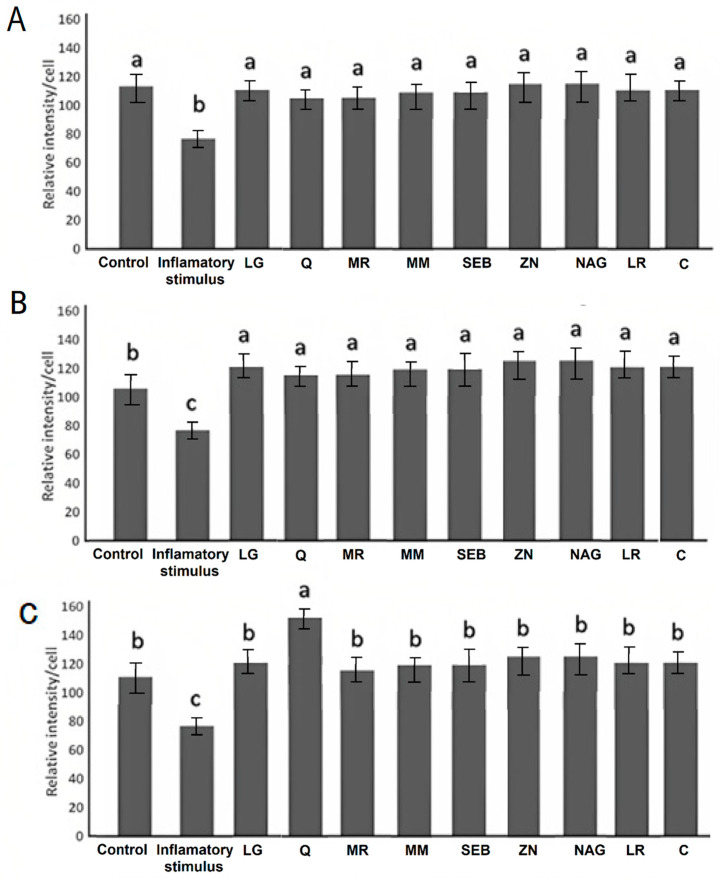
(**A**) ZO-1, (**B**) occludin, and (**C**) claudin-1 relative intensity/cell of control (healthy and untreated cells with no inflammatory stimulus) and inflammatory stimulus (IS) (cells treated with only IL-1β, TNF-α, IFN-γ, LPS, and isoflavone genistein) yogurt samples: Q = quercetin, MR = marshmallow root, NAG = N-acetyl-D-glucosamine, LG = L-glutamine, ZN = zinc orotate, MM = maitake mushrooms, LR = licorice root, SEB = slippery elm bark, and C = control yogurt with IS. ^a–c^ Different letters denote significant differences between groups at *p* < 0.05 among control yogurt and C samples in one-way ANOVA followed by Tukey test.

The disruption of the intestinal epithelium in leaky gut-related disorders is due to a combination of genetic and environmental factors that unbalance the proliferation and death of epithelial cells. Immunofluorescence microscopy images of Caco-2 cells are overlaid with antibodies ZO-1 (Figure 5), occludin-1 (Figure 6), and claudin-1 (Figure 7). These images show a continuous green pattern of tight junctions between cells. The fluorescence intensity for ZO-1 (Figure 5), occludin-1 (Figure 6), and claudin-1 (Figure 7) is seen in the net green shape patterns between cells. Red arrows shown in Figure 5C, Figure 6C, and Figure 7C between cells indicate tight junctions in a healthy (control) cell network, as reported by Putt et al. (2017) [33] and Li et al. (2004) [40]. On the contrary, ZO-1 (Figure 5), occludin-1 (Figure 6), and claudin-1 (Figure 7) had lower fluorescence intensity (Figure 8) in Caco-2 cells treated with only the mixture for induction when compared to the healthy (control) cells.

Yogurt samples (Q, MR, NAG, LG, ZN, MM, LR, SEB, and CY) with lipopolysaccharide, interleukin-1β, tumor necrosis factor-α, interferon-γ, and isoflavone genistein increased the fluorescence intensity (by visual observation) of ZO-1 (Figure 5), occludin-1 (Figure 6), and claudin-1 (Figure 7). Probiotics including *Lactobacillus rhamnosus* GG, *Lactobacillus acidophilus*, *Lactobacillus plantarum*, *Bifidobacterium infantis*, *Bifidobacterium animalis* subsp. *lactis* BB-12, and *Escherichia coli Nissle* 1917 have been shown to exert a function in modulating intestinal barrier functions by increasing tight junctions [2]. Probiotics should be considered a vital part of the holistic healing of leaky gut. This is because they work simultaneously to restore the intestinal environment while helping to rebalance the intestinal microbiota. Gut dysbiosis is an overgrowth of harmful bacteria and organisms in the intestine. Results from several studies suggest a connection between dysbiosis and leaky gut. Since an imbalance of intestinal flora can likely cause leaky gut, we can infer that probiotics help cure intestinal permeability [2]. For claudin-1 (Figure 7), the fluorescence intensity (Figure 8) of yogurt fortified with quercetin was the highest among all yogurt samples (MR, NAG, LG, ZN, MM, LR, SEB, and CY). Amasheh et al. (2008) [41] reported that quercetin could upregulate claudin-1, claudin-3, claudin-4, and claudin-7 in Caco-2 cells. Immunofluorescence microscopy pictures (Figure 5, Figure 6 and Figure 7) indicated similar results to the paracellular permeability and TEER observations, where the integrity of Caco-2 cells with yogurt samples incorporating quercetin was the highest among all treatments. Uniform tight junctions forming between adjacent cells indicate fluorescence intensity in healthier cells. Understanding the mechanisms of tight junction modulation is crucial for developing functional foods. Probiotics can decrease chlorine and water secretion induced by enteroinvasive *E. coli*. It has been proposed that they modify the expression of tight junction proteins. It has been shown that probiotics reduce lesions caused by pathogenic strains of *Escherichia* [2]. Nevertheless, more research is required to elucidate the mechanism by which yogurt fortified with functional ingredients participates in the repair process of intestinal epithelium tight junctions.

### 2.6. Tight Junction Expression Analysis

**Figure 9:**
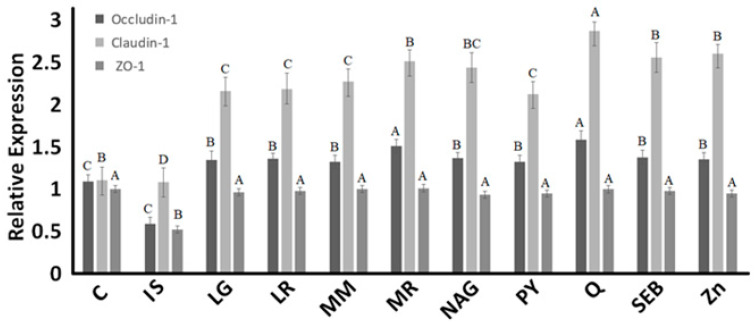
Relative expression of ZO-1, claudin-1, and occludin in different yogurt samples with growth media (C) and inflammation stimulus (IS). Yogurt samples were treated with IS and the following ingredients: LG = L-glutamine, LR = licorice root, MM = maitake mushrooms, MR = marshmallow root, NAG = N-acetyl-D-glucosamine, PY = control yogurt, Q = quercetin, SEB = slippery elm bark, and Zn = zinc orotate. ^ABC^ Occludin relative expression means across the various ingredients not containing a common letter were significantly different (*p* < 0.05). ^ABCD^ Claudin-1 relative expression means across the various ingredients not containing a common letter were significantly different (*p* < 0.05). ^AB^ ZO-1 relative expression means across the various ingredients not containing a common letter were significantly different (*p* < 0.05) in one-way ANOVA followed by Tukey test.

Caco-2 cells are the most striking example of cellular differentiation that exists. Their enterocyte-type differentiation is characterized by the organization of the cells in a cell monolayer, with the presence of tight junctions in the apical part of the intercellular space, and a brush border with a cytoskeleton composed of actin filaments associated with specific proteins such as villin. This brush border of the cells expresses intestinal hydrolases and glucose and amino acid transporters. Real-time quantitative polymerase chain reaction was utilized to study whether yogurt enriched with the ingredients enhanced the gene expression of ZO-1, claudin-1, and occludin in the Caco-2 cell model. The relative expression of ZO-1, claudin-1, and occludin is shown in Figure 9. When Caco-2 cells were treated with only an inflammatory stimulus (mixture for induction) (lipopolysaccharide, tumor necrosis factor-α, interferon-gamma, and interleukin-1β), the relative expression of occludin, ZO-1, and claudin-1 decreased when compared to the control (cells treated with growth medium only (C)). The levels of ZO-1 relative expression for yogurt samples did not differ compared to the control cells (healthy cells). Compared to the control, the relative expression levels of claudin-1 and occludin increased in yogurt fortified with marshmallow root and quercetin.

The increase in the expression levels of claudin-1 and occludin in yogurt enriched with marshmallow root and quercetin and the inflammatory stimulus was associated with the observations of transmission electron and immunofluorescence microscopy (Figure 4, Figure 5, Figure 6, Figure 7 and Figure 8), where tight junctions were retained mainly by yogurts incorporating quercetin. Furthermore, the paracellular permeability (Table 2) and transepithelial electrical resistance (Figure 2) measurements show that yogurt fortified with marshmallow root and quercetin decreases intestinal barrier dysfunction by possibly improving the tight junction proteins, as shown via transmission electron microscopy (Figure 4) and immunofluorescence microscopy (Figure 5, Figure 6, Figure 7 and Figure 8). The gastrointestinal tract constitutes the main surface of exchange and communication between the external environment and the internal environment. In the adult individual, the gastrointestinal mucosa is endowed with structures and functions specifically adapted to the recognition of substances that pass through the digestive tract [2]. Probiotics can affect multiple signaling pathways of the intestinal epithelium that modulate the integrity of TJs, and the maintenance and restoration of barrier function, including those of the Rho family GTPases, PKC and MAPK [2]. It is important to take into account the diverse and complex interactions between the different biological and biochemical components of the intestinal barrier in the development of strategies to improve the integrity of the barrier [2].

### 2.7. Transepithelial Electrical Resistance Values in Digested Yogurt

**Figure 10:**
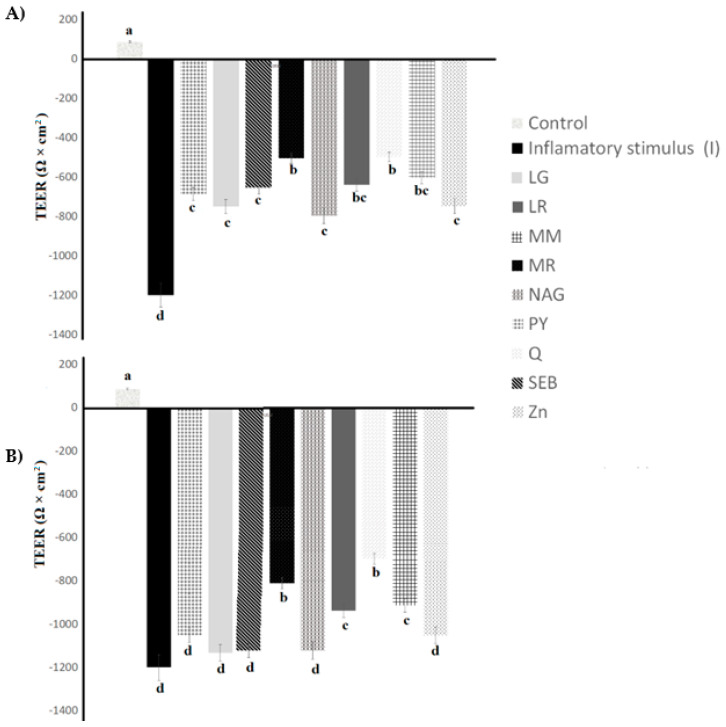
In vitro simulated (**A**) gastric digestion and (**B**) intestinal digestion of yogurt. Differentiated Caco-2 cells were treated with a vehicle control (C), inflammatory stimulus (I) (25 ng mL^−1^ IL-1β, 50 ng mL^−1^ TNF-α, 50 ng mL^−1^ IFN-γ, and 1 μg mL^−1^ LPS) 0.03 g mL^−1^, and yogurt samples for 48 h. Yogurt samples were treated with IS and the following ingredients: LG = L-glutamine, LR = licorice root, MM = maitake mushrooms, MR = marshmallow root, NAG = N-acetyl-D-glucosamine, PY = control yogurt, Q = quercetin, SEB = slippery elm bark, and Zn = zinc orotate. Values are means ± SD, with n = 7 for each treatment. ^abcd^ Means not containing a common letter were significantly different, as determined via one-way ANOVA followed by Tukey-HSD test (*p* < 0.05).

The transepithelial electrical resistance (TEER) of digested yogurt samples, as influenced by the incorporation of the ingredients over 48 h of incubation, is illustrated in Figure 10. For gastric digestion, the TEER values followed a similar trend to the undigested yogurt samples (Figure 10), where Q and MR had the highest TEER values for undigested yogurt, as well (Figure 2). The TEER of yogurt samples incorporating quercetin and marshmallow root was higher than the control yogurt sample of the undigested yogurt samples and yogurt with gastric digestion. The gastric digestion of samples did not affect the inflammatory disruption of the TEER values (Figure 10A). Putt et al. (2017) [33] reported that gastric digestion did not affect the TEER values of low-fat yogurt.

On the other hand, the TEER values were significantly higher for yogurt samples made with quercetin, maitake mushroom, licorice root, and marshmallow root compared to the control yogurt samples for intestinal digestion (Figure 10B).

Digested yogurt loses bioavailability during intestinal digestion, and the active protein and/or peptide bioavailability could be lost upon treatment with pancreatin. Alginate-milk microspheres can encapsulate *L. bulgaricus* and increase survivability for 1 and 2 h in 1% and 2% porcine bile salt solutions [42]. In maitake mushroom, licorice root, and marshmallow root, polysaccharides with encapsulating properties have been reported [43,44,45]. It is possible that polysaccharides in these ingredients could have an encapsulating effect on probiotic bacteria peesnet in yogurt, aid in producing active proteins and/or peptides, and improve bioavailability in intestinal digestion. Thus, they also inhibit the enzymatic activity of the enzymes presented and protein digestion in the intestinal phase, since some peptides from the yogurt have shown inflammatory properties. Quercetin has been shown to inhibit protein digestion in the digestive tract with the intestinal fluid by inhibiting trypsin [46]. It is possible that quercetin could improve the bioavailability of yogurt’s active proteins and/or peptides.

## 3. Materials and Methods

### 3.1. Ingredients

Marshmallow root (Grassroots Herb Supply, Arden Hills, MN, USA), slippery elm bark (iherb, Moreno Valley, CA, USA), quercetin (Bulk Supplements, Henderson, NV, USA), L-glutamine (Bulk Supplements, Henderson, NV, USA), NAG (Bulk Supplements, Henderson, NV, USA), maitake mushrooms (Natural Foods, West Palm Beach, FL, USA), zinc orotate (Bulk Supplements, Henderson, NV, USA), licorice root (Banyan Botanicals, Williams, OR, USA), and starter cultures of and Lactobacillus delbrueckii subsp. bulgaricus and *Streptococcus thermophilus* (Chr. Hansen, Milwaukee, WI, USA) were purchased. Milk was obtained from Kleinpeter Farms Dairy (Baton Rouge, LA, USA).

### 3.2. Experimental Design

Plain yogurts were prepared from the source milk, to which eight different ingredients were randomly assigned. The eight ingredients were quercetin (700 mg/L), marshmallow root (1340 mg/L), L-glutamine (7 mg/L), NAG (210 mg/L), zinc orotate (70 mg/L), maitake mushrooms (42 mg/L), slippery elm bark (210 mg/L), and licorice root (210 mg/L) [47]. The control did not contain any ingredients. TEER was analyzed at 0, 24, 48, and 72 h, whereas paracellular permeability was examined at 0, 5, 10, and 15 h. Tight junction expression, immunofluorescence microscopy, and TEM were performed after 2 days of storage. Three replications were conducted.

### 3.3. Yogurt Preparation

Cows’ milk was obtained from Kleinpeter Farms Dairy (Baton Rouge, LA, USA). Milk composition was 86.5% water, 5% carbohydrate, 3.5% fat, 4.3% protein, and 0.7% minerals. Milk was divided equally into nine pails (11.36 L per pail), and the eight ingredients were randomly assigned to these eight pails, including the control group (pail with no ingredients). Milk with ingredients was pasteurized in a VEVOR stainless steel pail can by heating it for 30 min at 85 °C. The milk mix was periodically stirred vigorously using a commercial immersion blender (Waring Commercial, McConnellsburg, PA, USA). After pasteurization, the milk mix was tempered to 41 °C, inoculated with *Lactobacillus bulgaricus* LB-12 and *Streptococcus thermophilus* ST-M5, and blended. The obtained blend was poured into 355 mL plastic cups (Alcoa, Inc., Pittsburgh, PA, USA) and incubated (41 °C) until the pH reached 4.6. The yogurt samples were stored at 4 °C for 1 day before freeze-drying the samples [48].

### 3.4. Simulated Gastric and Intestinal Digestion of Yogurt

The control yogurts and yogurts fortified with ingredients were subjected to simulated in vitro gastric and intestinal digestion described by Minekus et al. (2014) [49] with minor changes. For gastric digestion, lyophilized yogurt (0.2 g) was mixed with 4 mL of 0.15 N HCl, and then, manufactured gastric fluid (Chemazone, Edmonton, Alberta, Canada) was mixed at a proportion of 1:1 (*w*/*v*). The solution was then mixed with porcine pepsin enzyme (Sigma-Aldrich, St. Louis, MO, USA) (2000 U mL^−1^). The solution was then set to a pH of 3 with 0.1 N HCl and was incubated for 3 h at 37 °C with continuous mixing. For the intestinal phase, the solution obtained from gastric digestion was mixed with simulated gastric fluid (Chemazone, Edmonton, Alberta, Canada) at a proportion of 1:1 (*v*/*v*). The solution was then mixed with pancreatin enzyme (Sigma-Aldrich, St. Louis, MO, USA) (100 U mL^−1^) and oxgall bile salt (US Biological, Swampscott, MA, USA) (10 mM). The solution was then set to a pH of 7 with 0.1 N HCl, and the solution was incubated for 7 h at 37 °C with continuous mixing. All samples were immediately collected using liquid nitrogen after the in vitro digestion process via snap freezing.

### 3.5. Diphenyl-2-picrylhydrazyl (DPPH) Radical Scavenging Assay

The DPPH test was conducted similarly to the method presented by Najgebauer-Lejko et al. (2011) [50] with small modifications. The yogurt was lyophilized (Labconco Free zone, Kansas City, MO, USA) for ~72 h. The freeze-dried yogurt (600 mg) was combined with 40 mL of 80% methanol solution in a 60 mL centrifuge tube. The solution was stirred, shaken, and sonicated (100 W; 15 min), and the solution was then centrifuged at 1700× *g* force for 8 min. The yogurt extract (supernatant) was used for antioxidant activity examinations. A total of 100 μL of yogurt extracts were added to 3.0 mL of 0.1 mM DPPH reagent, and the obtained solution was incubated in the dark at 25 °C for 2 h. Absorbance was recorded using a spectrophotometer (Genesys SEC10UV, Thermo Fisher, Waltham, MA, USA) at 515 nm. Distilled water was used as the blank, and the methanol-DPPH reagent solution (100:3.9) was used as the control (3.9 mL). The radical scavenging activity was estimated as described in Equation (1):

% Inhibition = (Absorbance control − Absorbance sample/Absorbance control) × 100 (1)

### 3.6. Ferric Reducing Antioxidant Potential (FRAP) Assay

The FRAP radical scavenging test was conducted similarly to the method illustrated by Benzie and Strain (1996) [51] with minor modifications. A total of 200 μL of yogurt extracts (as described above) were combined with 1.8 mL of ferric reducing antioxidant potential reagent (Sigma Chemical Co., St. Louis, MO, USA), and the obtained solution was incubated in a water bath (37 °C; 10 min). The FRAP reagent consisted of 8 mM 2,4,6-tri(2-pyridyl)-s-triazine (TPTZ) reagent (Sigma Chemical Co., St. Louis, MO, USA), 300 mM acetate buffer, and 20 mM FeCl_3_ (Sigma Chemical Co., St. Louis, MO, USA) at a proportion of 1:10:1. The absorbance was recorded utilizing a spectrophotometer (Genesys 10UV, Thermo Fisher, Waltham, MA, USA) at 593 nm. FeSO_4_·7H_2_O (0.3–1.0 mM) (Sigma Chemical Co., St. Louis, MO, USA) was used for the calibration curve, and the results are reported as mmol Fe_2_+ equivalent/l (mmol Fe_2_+ E/L).

### 3.7. Ferrous Ion Chelating (FIC) Assay

The FIC radical scavenging test was performed using the method presented by Chan et al. (2007) [52]. Yogurt extracts were mixed with 0.1 mM iron (II) sulfate hydrate (FeSO_4_·xH_2_O) solution (Sigma Chemical Co., St. Louis, MO, USA) and 0.25 mM ferrozine solution (Sigma Chemical Co., St. Louis, MO, USA) at a ratio of 1:10:1. The obtained solution was incubated at 25 °C for 10 min. The absorbance was estimated using a spectrophotometer (Genesys 10UV) at 562 nm. Distilled water was used as the blank, and the FeSO_4_·xH_2_O (1 mL), ferrozine (1 mL), and distilled water (1 mL) solution was used as the control. The FIC capacity was calculated as illustrated in Equation (1).

### 3.8. Caco-2 Cell Culture Maintenance

Caco-2 cells were obtained from the American Type Culture Collection—ATCC (Manassas, VA, USA). The cells were seeded in T25 (SPL) flasks at a concentration of 5 × 10^5^ cells per flask with 7 mL of high-glucose DMEM (Life Technologies, Carlsbad, CA, USA) culture medium, supplemented with 10% inactivated FBS (Life Technologies, Carlsbad, CA, USA), L-glutamine (Life Technologies, NY, USA) 1%, and penicillin–streptomycin mixture 1% (Life Technologies, Carlsbad, CA, USA). Cells were incubated at 37 °C with 5% CO_2_, 95% air, and 90% relative humidity to 90% confluency. The medium was replaced every 3 days. For permeability studies, cells from cultures in T25 flasks (6 × 10^4^ cells/cm^2^) were seeded on Transwell^®^ polyethylene terephthalate (PET) inserts with medium changes every three days. The layers obtained were observed via inverted microscopy, and the growth speed was established as the time required for each strain to reach confluence. The Caco-2 cells were differentiated for 21 days. Caco-2 cells were subculture with trypsin (2 × 10^5^ cells per mL) onto 0.4 μm polycarbonate membrane Transwell inserts (Corning, Inc.; Lowell, MA, USA) [53].

### 3.9. Caco-2 Cell Viability Test

Caco-2 cells were subcultured at 10^3^ per well on 96-well plates with media integrated with powdered yogurt (control yogurt). Yogurt powder was resuspended in growth media at 1:25, 1:50, 1:75, and 1:100 dilution ratios before its application to cell monolayers. The cells were incubated for 0, 24, and 48 h at 37 °C with 5% CO_2_. The highest concentration of nontoxic yogurt–water solution was used for subsequent analysis in the Caco-2 cells. Caco-2 cells were treated with phosphate-buffered saline (PBS) and considered the negative control. Cells were washed according to the CellTiter 96 Aqueous One solution (Promega, Madison, WI, USA) protocol with tetrazolium compound and phenazine methosulfate solution. Cell density was measured using a BioRad Model 680 microplate reader at a 490 nm wavelength. Formazan was used as an indicator to estimate the number of living cells. TEER was also measured after 48 h to examine the effect of powdered yogurt dilutions (1:25, 1:50, 1:75, and 1:100) on differentiated Caco-2 integrity. The cell viability assay and TEER measurements were performed in triplicate within cell batches and with 6 measurements within 1 cell batch. 

### 3.10. Induction of Barrier Dysfunction in Caco-2 Cells

The Caco-2 cells were treated with lipopolysaccharide (LPS, 1 μg mL^−1^) and cytokines such as interleukin-1β (IL-1β, 25 ng mL^−1^), tumor necrosis factor-α (TNF-α, 50 ng mL^−1^), and interferon-gamma (IFN-γ, 50 ng mL^−1^) (mixture for induction). The cytokines were applied in the basolateral compartment with supplemented growth media, whereas lipopolysaccharide was incorporated into the basolateral and apical sections. Isoflavone genistein, known to have inflammatory properties, was utilized as a positive [33].

### 3.11. Transepithelial Electrical Resistance (TEER)

TEER was measured using a Millicell-ERS device (Millipore Corp., Bedford, MA, USA). The measurements were carried out. TEER values were obtained for the inserts with cells in transport buffer (BT, 25 mM HEPES in HBSS); the value recorded in an insert with BT and without cells was subtracted. The result was multiplied by the effective membrane area to obtain the final TEER value. Only those monolayers with TEER values greater than 550 Ω·cm^2^ were applied [33]. All experiments were conducted in triplicate. TEER of digested samples was treated as undigested yogurt.

### 3.12. Paracellular Permeability

Paracellular permeability was evaluated as in Chelakkot et al. (2018) [54] and Mohebali et al. (2020) [55] by estimating the permeability of Lucifer yellow (LY) and fluorescein isothiocyanate (FITC)–dextran 4000 (FD) through the cell monolayers. LY (0.5 mg mL^−1^) and FD (1 mg mL^−1^) were added to HBSS/HEPES solution at 37 °C, and the HBSS/HEPES solution with FD and LY (0.2 mL) was incorporated into the apical section. In addition, 1 mL of HBSS was incorporated into the basolateral section. The flux was measured in six inserts, and a 25 mM solution of AL (GIBCO) in BT was added to the apical chamber in each Transwell^®^ with shaking at 50 rpm for 120 min at 37 °C, 90% relative humidity, and 5% CO_2_. Samples were removed from the basolateral chamber at 0, 4, 8, 12, and 16 min, and the addition of the same volume of BT compensated for the volume removed. Readings were performed using a SPECTRAmax GEMINI XS spectrofluorometer at excitation wavelengths of 428/540 nm (LY) and 485/530 nm (FD). The apparent permeability coefficient (Papp (cm/s) was calculated using Equation (2):

P_app_ (cm s^−1^) = dQ/dt × 1/(A × C) (2)

where C is the initial amount of fluorescent marker on the apical section (mol mL^−1^), dQ is the concentration (fluorescent marker) on the basolateral section (mol mL^−1^), A is the membrane surface area (cm^2^), and dt is the flux per second (1/s).

### 3.13. Transmission Electron Microscopy (TEM)

The interaction between yogurt treatments (Q, MR, NAG, LG, ZN, MM LR, SEB, and CY) and Caco-2 cells (1 µm) was examined via transmission electron microscopy; the inflammatory stimuli were isoflavone genistein, lipopolysaccharide (LPS), interferon-gamma (IFN-γ), tumor necrosis factor-α (TNF-α), and interleukin-1β (IL-1β) (mixture for induction). Samples with inflammatory stimuli were considered negative controls. After dehydration, the cells were gathered using glass knives on an ultramicrotome (Reichert Ultracut S, Depew, NY, USA), inserted in Araldite epoxy resin, and colored with toluidine blue 1% in 1% sodium borate solution (Sigma-Aldrich, Germany). Ultrathin sections (90 nm) were cut using a DiATOME diamond knife (Reichert, Wien, Austria) and stained with a heavy metal stain solution of uranyl acetate solution and lead citrate. Ten clear spots per sample were observed using a TEM microscope (Hitachi H7000, 100 kV, Yokohama, Japan).

### 3.14. Immunofluorescence Light Microscopy

The immunofluorescence microscopy procedure was performed according to Zeng et al. (2016) [56] with slight changes. The cells (4 × 10^5^ cells/cm^2^) were treated using a Lab-Tek II chamber slide system (Nalge Nunc International) with control media, inflammatory stimulus (IS), and yogurt samples (Q, MR, NAG, LG, ZN, MM LR, SEB, and CY) with IS. Caco-2 cells were rinsed with Hank’s balanced salt solution with 3% paraformaldehyde and without Mg and Ca. They were mounted on a previously gelatinized slide and stored at −80 °C until staining. The slides with the sections were washed with PBS and fixed with 10% neutral formalin for 15 min and at room temperature. They were then washed with PBS and permeabilized for one hour with Triton x-100 (0.25% in PBS). Cells were overlaid (diluted at 1:50) with the antibodies ZO-1, occludin-1, and claudin-1 (Zymed Laboratories, San Francisco, CA, USA). The ZO-1, occludin-1, and claudin-1 staining of Caco-2 cell monolayers was conducted according to Putt et al. (2017) [33] and Yokoo et al. (2021) [57]. Cells were washed and blocked for 35 min with 1.5% (*w*/*v*) bovine serum albumin in PBS for ZO-1, occludin-1, and claudin-1. The occludin antibody was incubated overnight at 4 °C, and the ZO-1 antibody was incubated for 4 h at 37 °C for ZO-1, as Putt et al. (2017) [33] described. Claudin-1 was incubated for 16 h at 4 °C [57]. After the incubation of ZO-1 and Occudin-1, goat anti-rabbit IgG antibody (Sigma) and (H+L) FTIC conjugate (Sigma) were integrated at a ratio of 1:100 to the Caco-2 cell monolayer and incubated for 30 min (ZO-1 and occudin-1) and 1 h (claudin-1). Fluorescence was monitored using a fluorescent light microscope and FITC-compatible media (Compound Microscope Leitz Optilux, Germany). Immunofluorescence pictures were obtained using ZEN 2010 software (Carl Zeiss AG, Oberkochen, Germany). All tight junctions were viewed between 10 and 15 fields under balanced staining [33]. The image size obtained was 80 μm^2^.

### 3.15. Gene Expression Analysis of Tight Junction Proteins

Cell RNA extraction with slight changes was conducted using Popović et al. (2020) [58]. Using an Ambion DNA-freeTM Kit (Thermo Fisher Scientific, Waltham, MA, USA), Caco-2 cells (5 × 10^6^ cells) were collected with a DNaseI and centrifuged at 3000× *g*. The reverse transcription verification was performed using the RevertAid RT kit (Thermo Fisher Scientific, Waltham, MA, USA). Via fluorometric quantitation, Qubit (Thermo Fisher Scientific, Waltham, MA, USA) was used to determine RNA levels. A total of 1 µg of isolated RNA was reverse transcribed to cDNA at a 15 mL reaction volume via 7500 real-time PCR using the RevertAid First Strand cDNA Synthesis Kit (Thermo Scientific). Real-time quantitative polymerase chain reaction (RT-qPCR) was conducted using iTaq™ Universal SYBR-Green Supermix on a Bio-Rad CFX96 system following the method described by the supplier. The primers for occludin (forward: CCA ATG TCG AGG AGT GGG, reverse: CGC TGC TGT AAC GAG GCT), claudin-1 (forward: TGG TGG TTG GCA TCC TCC TG, reverse: AAT TCG TAC CTG GCA TTG ACT GG), ZO-1 (forward: CAA GAT AGT TTG GCA GCA AGA GAT G, reverse: ATC AGG GAC ATT CAA TAG CGT AGC), ribosomal protein large P0 (forward: CTC GTG GAA GTG ACA, reverse: TCG TCT GCT TGG AGC CCA CAT TGT CT), and 18s RNA (RNA18S5) (forward: CTG AGA AAC GGC TAC CAC ATC, reverse: GCC TCG AAA GAG TCC TGT ATT G) were selected according to Putt et al. (2017). According to Maubon et al. (2007) [59] and Vreeburg et al. (2011) [60], the gene expression was normalized and standardized to a mathematic mean using RNA18S5 and RPLP0.

### 3.16. Statistical Analysis

All experiments were conducted in triplicate, and the data were processed via SAS (Statistical Analysis Systems) (SAS Institute Inc., Cary, NC, USA) using ANCOVA PROC GLM for cell viability examinations, paracellular permeability observations, and transepithelial electrical resistance determinations, and where differences in least square means were used to determine significant differences at *p* < 0.05 for the main effect (ingredients vs. control), time effect, and interaction effect (treatments * time). In addition, ANOVA was used, followed by Tukey’s test for paracellular permeability coefficients, antioxidant capacity measurements, immunofluorescence light microscopy observations, and gene expression analysis.

## 4. Conclusions

The influence of yogurts supplemented with L-glutamine, quercetin, slippery elm bark, marshmallow root, N-acetyl-D-glucosamine, licorice root, maitake mushrooms, and zinc orotate on intestinal barrier dysfunction was examined. Yogurt with quercetin and marshmallow root enhanced the antioxidant activity and the integrity of the Caco-2 cell monolayer by increasing tight junctions. Gastric digestion did not affect inflammation-induced Caco-2 barrier dysfunction. In contrast, the intestinal digestion of yogurt negatively influenced the integrity of the Caco-2 barrier. Nevertheless, yogurt fortification with quercetin, marshmallow root, maitake mushroom, and licorice root improved the TEER values compared to the control yogurt. Yogurt supplementation with quercetin, marshmallow root, maitake mushroom, and licorice root may enhance intestinal barrier function. It could be an option to consider when formulating a product for consumers with leaky gut. Nevertheless, more studies in vivo are needed to confirm these results and determine the mechanisms of action. Furthermore, the fortification of these ingredients into yogurt should also be confirmed in clinical trials for future studies. Probiotics are a vital approach to healing leaky gut. They should be used in conjunction with an anti-inflammatory diet for the best results. Probiotics help rebalance the intestinal flora, restore the intestinal wall, and reduce inflammation.

## Figures and Tables

**Table 1:** Antioxidant potential of yogurt containing ingredients, as measured by assessing DPPH (1, 1-diphenyl-2-picrylhydrazyl) radical scavenging activity, FRAP (ferric reducing antioxidant potential) and FIC (ferrous ion chelating) ability.

Sample	DPPH Radical Scavenging Activity (%)	FRAP (mmol Fe^2+^ E/L)	FIC Ability (%)
Control	94.03 ± 0.45 ^b^	20.11 ± 1.45 ^bc^	80.93 ± 0.45 ^b^
LG	94.37 ± 0.67 ^b^	21.29 ± 1.76 ^bc^	80.54 ± 0.24 ^b^
SEB	93.67 ± 0.59 ^b^	21.16 ± 1.23 ^bc^	80.32 ± 0.63 ^b^
ZN	94.42 ± 0.77 ^b^	19.47 ± 1.65 ^c^	80.30 ± 0.44 ^b^
NAG	93.59 ± 0.29 ^b^	21.27 ± 1.05 ^bc^	80.71 ± 0.37 ^b^
LR	94.83 ± 0.50 ^b^	20.75 ± 1.32 ^bc^	80.22 ± 0.55 ^b^
MM	93.59 ± 0.37 ^b^	22.34 ± 1.54 ^b^	80.49 ± 0.76 ^b^
MR	96.56 ± 0.89 ^a^	23.67 ± 1.34 ^ab^	83.15 ± 0.47 ^a^
Q	97.18 ± 0.29 ^a^	25.45 ± 1.62 ^a^	84.55 ± 0.58 ^a^

^abc^ Column means not containing a common letter are significantly (*p* < 0.05) different in one-way ANOVA followed by Tukey test. LG = L-glutamine, SEB = slippery elm bark, ZN = zinc orotate, NAG = N-acetyl-D-glucosamine, LR = licorice root, MM = maitake mushrooms, MR = marshmallow root, and Q = quercetin.

**Table 2:** Apparent permeability coefficients (Papp) for fluorescein isothiocyanate–dextran (FD) and Lucifer yellow (LY) after treatment of Caco-2 cells for 48 h with the control (C), an inflammatory stimulus (I), or inflammatory stimulus with yogurt (1:25, *w*/*v*) with ingredients.

Sample	FD_Papp_ (×10^−7^ cm/s)	LY_Papp_ (×10^−7^ cm/s)
Inflammatory stimulus (I)	7.03 ± 1.37 ^c^	11.04 ± 1.84 ^a^
Growth media	4.22 ± 1.66 ^b^	7.27 ± 1.45 ^b^
Control yogurt	4.37 ± 0.67 ^b^	8.04 ± 1.26 ^b^
LG	3.72 ± 1.58 ^b^	8.48 ± 1.65 ^b^
SEB	4.42 ± 1.45 ^b^	8.55 ± 1.83 ^b^
ZN	3.67 ± 1.56 ^b^	9.04 ± 1.59 ^b^
NAG	4.03 ± 1.19 ^b^	9.11 ± 2.05 ^b^
LR	3.33 ± 1.87 ^b^	8.62 ± 1.96 ^b^
MM	3.28 ± 1.73 ^ab^	9.18 ± 2.07 ^b^
MR	2.54 ± 1.11 ^a^	7.67 ± 1.38 ^b^
Q	2.77 ± 1.45 ^a^	6.45 ± 1.77 ^b^

^abc^ Column means not containing a common letter are significantly (*p* < 0.05) different in one-way ANOVA followed by Tukey test. LG = L-glutamine, SEB = slippery elm bark, ZN = zinc orotate, NAG = N-acetyl-D-glucosamine, LR = licorice root, MM = maitake mushrooms, MR = marshmallow root, and Q = quercetin.

## Data Availability

Data is contained within the article.
